# TRAF3IP3 promotes glioma progression through the ERK signaling pathway

**DOI:** 10.3389/fonc.2022.776834

**Published:** 2022-09-16

**Authors:** Qi Lin, Zhen Chen, Zhao-Li Shen, Fei Xue, Jia-Jun Qin, Xi-Peng Kang, Zhong-Rong Chen, Zhong -Yuan Xia, Liang Gao, Xian-Zhen Chen

**Affiliations:** ^1^ Department of Neurosurgery, Shanghai Tenth People’s Hospital, Tongji University School of Medicine, Shanghai, China; ^2^ Tongji University School of Medicine, Shanghai, China

**Keywords:** TRAF3IP3, glioma, prognosis, ERK, differentially expressed gene

## Abstract

TRAF3IP3 was reportedly associated with poor prognosis in patients with melanoma; however, its role in glioma is unknown. We aimed to demonstrate the relationship between TRAF3IP3 and glioma and to investigate the potential role of TRAF3IP3 in glioma. Datasets were collected from The Cancer Genome Atlas (TCGA) and Gene Expression Omnibus (GEO) databases. We used the Wilcoxon rank-sum test to compared TRAF3IP3 expression in normal and glioma tissues. Kaplan–Meier analysis was performed to evaluate the correlation between TRAF3IP3 and patient survival rate. Gene set enrichment analysis (GSEA) was used to annotate the biological function of TRAF3IP3 in glioma. We also examined the effects of TRAF3IP3 on glioma progression, including characteristics such as cell proliferation, migration, and invasion, using cell proliferation, wound healing, and Transwell assays, respectively, paired with *in vitro* glioma cell lines and *in vivo* mouse xenograft models to determine the molecular mechanisms underlying these effects. High TRAF3IP3 expression in glioma tissues was associated with patients with neoplasm cancer tissue source site, and poorer overall survival (OS) (p = 0.03), which was validated using TCGA. GSEA revealed the enrichment of neuroactive ligand–receptor interactions, the olfactory pathway, proteasome pathway, cytokine–cytokine receptor interactions, and calcium signaling pathway in the TRAF3IP3 high-expression phenotype. TRAF3IP3 knockdown markedly suppressed the proliferation, migration, and invasion abilities of U251 glioma cells, whereas TRAF3IP3 overexpression notably promoted the progression of U118 cell tumors. Mechanistic studies revealed that TRAF3IP3 upregulated p-ERK expression in glioma cells. Notably, the ERK signaling pathway inhibitor U0126 drastically attenuated the effects of TRAF3IP3 on p-ERK and markedly blocked its tumor-promoting activity. TRAF3IP3 overexpression also promoted *in vivo* tumor growth in a nude mouse xenograft model. Collectively, TRAF3IP3 stimulates glioma cell proliferation, migration, and invasion, at least partly by activating the ERK signaling pathway. We hypothesize that TRAF3IP3 may participate in glioma development *via* the ERK signaling pathway and that elevated TRAF3IP3 expression may serve as a potential biomarker for glioma prognosis.

## Introduction

Glioma is one of the top three malignant brain tumors in adults (1, 2). The tumor originates from neuroepithelial tissue and is characterized by high proliferation, invasion, and metastasis (3). The prognosis of patients with glioma is extremely poor, with an average survival rate of only 14.4 months (4). After surgery, cancer recurs in most patients within 6 months, posing a great challenge to the clinical treatment of gliomas (5). The development of chemotherapy drugs, such as temozolomide, has prolonged the survival of patients; however, drug resistance remains an issue (6). In addition, there are no reliable and accurate biomarkers for identifying the early stages of glioma. Therefore, it is necessary to identify new tumor progression-related biomarkers and new targets for the treatment and prognostic evaluation of patients with gliomas (7).

TRAF3IP3, also known as TRAF3-interacting Jun N-terminal kinase-activating modulator (T3JAM), was first identified in 2003. It can specifically interact with tumor necrosis factor receptor-associated factor 3 (TRAF3). The combination of TRAF3IP3 and TRAF3 can form a detergent-insoluble complex that mediates the activation of the JNK signaling pathway. Previous studies have shown that TRAF3IP3 is mainly involved in T cell immunity and antiviral infection (8). It also reportedly plays a role in tumor development (9). Recently, it was reported that TRAF3IP3 is highly expressed in melanoma and closely related to tumor proliferation, invasion, and metastasis. Interestingly, higher expression levels of TRAF3IP3 in patients with melanoma predicted a poorer patient prognosis (10). However, TRAF3IP3 expression in gliomas and its correlation with patient prognosis have not been reported.

This study identified TRAF3IP3 expression profiles in glioma and its association with the disease to analyze its predictive value for the prognosis of patients with glioma. To investigate the differential TRAF3IP3 transcriptional and proteomic expression and clarify the potential prognostic value of TRAF3IP3 in patients with glioma, we analyzed gene expression profiles and clinical data from The Cancer Genome Atlas (TCGA) and various public databases, deciphering the underlying biological interaction networks, and the prognostic value of TRAF3IP3 gene expression. We confirmed that TRAF3IP3 regulated the proliferation, migration, and invasion of glioma cells *in vitro*. Silencing TRAF3IP3 expression significantly suppressed U251 cell proliferation compared to the controls. Fluorescence imaging experiments in nude mice showed a significant difference in tumor growth 21 days after the injection of TRAF3IP3-overexpressing glioma cells. TRAF3IP3 promoted glioma cell growth *in vivo*. In this study, we demonstrated that TRAF3IP3 promoted glioma proliferation by activating the ERK signaling pathway. U118 cells transfected with the PCDH/TRAF3IP3 vector were treated with or without the ERK pathway inhibitor U0126. We found that U0126 treatment significantly inhibited cell proliferation, migration, and invasion in PCDH/TRAF3IP3-transfected glioma cells and that the activation of the ERK signaling pathway mediated by TRAF3IP3 overexpression could be rescued by U0126. Our results indicate that TRAF3IP3 plays a key role in glioma progression.

## Materials and methods

### Data collection and differentially expressed genes

Gene expression profile data of glioma (GBM) GSE50161 (11) and GSE108474 (12) were downloaded from the Gene Expression Omnibus (GEO) database (13) using the R package GeoQuery and were divided into the tumor and normal tissue groups based on gene expression. The R package TCGAbiolinks (14) was used to obtain the gene expression profile and clinical data of patients with glioma from TCGA database. Detailed data on patients with GBM and the control groups are shown in **Table 1**.

**Table 1:** Basic clinical information sheet.

Clinical characteristic	Variable	Number of patients	*TRAF3IP3* expression	
			Low	High
Age	> =60	84	42	42
	< 60	76	39	37
Gender	Female	56	28	28
	Male	104	52	52
Status	Alive	51	26	25
	Dead	108	54	54
Race	WHITE	143	73	70
	BLACK OR AFRICAN AMERICAN	11	6	5
	ASIAN	5	3	2

The R package *limma* (15) was used to analyze differentially expressed genes (DEGs) in GBM tumor tissues relative to normal and adjacent tissues. In the three datasets, genes with adj p-value < 0.05 were deemed as DEGs, genes with log FC > 1 and adj p-value < 0.01 were considered upregulated genes, and genes with log FC < -1 and adj p-value < 0.01 were considered downregulated genes. We screened the intersection of DEGs of the three datasets to obtain the common DEGs.

### Function and pathway enrichment analysis

In this study, two functional enrichment analyses were used. The Gene Ontology (GO) function annotation analysis and Kyoto Encyclopedia of Genes and Genomes (KEGG) pathway enrichment analysis of the common DEGs were performed using the R software package clusterProfiler.

### Gene set enrichment analysis (GSEA)

GSEA (16) was performed using the GSEA package in R. Analysis was performed using default parameters. Functional and pathway enrichment analyses were performed on the DEGs in the high and low TRAF3IP3 expression groups.

### Construction of a protein–protein interaction (PPI) network

Using the Human Protein Reference Database (HPRD) to obtain the PPI network related to DEGs, we extracted the interaction subnets directly connected to at least 25 DEGs and visualized the obtained PPI network model with Cytoscape (17), using cytoHubba (18) to extract key subnets. Closely connected local regions in the single gene-related PPI network may represent molecular complexes and have specific biological functions, and ClueGO (19) was used for functional annotation.

### TRAF3IP3 expression grouping analysis

Patients were divided into high- and low-expression groups according to the TRAF3IP3 expression value in their tumor tissue. The difference between the TRAF3IP3 high-expression and low-expression groups was analyzed, and the prognosis of patients was evaluated at the gene expression level.

### Quantitative real-time PCR

Total cellular RNA was isolated from HMC3, U87, HS683, U251, and U118 cells using the TRIZOL reagent (15596-018, Invitrogen, USA), dissolved in RNase-free H_2_O, and stored at -80°C. cDNA synthesis was performed using M-MLV Reverse Transcriptase (AT341, TransGen, China) following the manufacturer’s instructions. The PCR system was used to amplify all transcripts using the AceQTM Universal SYBR Qpcr Master Mix (Q511-03, vazyme). A reaction mixture (total volume, 20 µL) was prepared using cDNA (8 μL), SYBR^®^ Green Master Mix (10 μL), upstream and downstream primers (0.5 μL), and ddH_2_O (1 μL). The qPCR was performed under the following conditions: pre-denaturation at 95°C for 5 min, denaturation at 95°C for 10 s, and annealing at 60°C for 30 s. Steps 2–3 were repeated for 40 cycles, and thereafter at 95°C for 15 s, at 60°C for 60 s, at 95°C for 15 s, and at 4°C. Target gene mRNA expression was verified and analyzed using the Illumina eco real-time PCR system using GAPDH as an internal standard. Quantification was performed by normalizing the expression levels of the target genes to the GAPDH expression levels; the expression levels of the genes were calculated using the 2^-ΔΔCt^ method. All experiments were performed in triplicate.

### Western blotting

For western blot analysis, cell lysates were prepared from cell lines or tissues with a RIPA lysis buffer kit (P0013B, Beyotime, China), and the protein concentrations were quantified using a Beyotime protein assay (P0010, Beyotime, China). Whole-cell proteins were separated using 10% sodium dodecyl sulfate-polyacrylamide gel electrophoresis (SDS-PAGE) and transferred to polyvinylidene difluoride membranes (Millipore, USA). The blots were blocked with 5% skim milk powder in TBST for 1 h at room temperature and incubated overnight with the following primary antibodies: TRAF3IP3 (ER65081, Huaan Bio, China, 1:1000), ERK (#4695, cst, 1:1000), p-ERK (#4370, cst, 1:2000), and GAPDH (#5174, 1:20000). Following three washes with TBST buffer, the membranes were incubated with secondary goat anti-rabbit (#7074, cst, 1:5000) or goat anti-mouse antibodies (#7076, cst, 1:5000) for 1 h at room temperature, followed by three washes with TBST buffer. Signals were detected using an enhanced chemiluminescence kit (Thermo, USA) and exposed to a film (4741023953, Guangxi Juxing Medical Instrument Co., Ltd). The protein density was normalized to that of GAPDH.

### Cell lines and cell culture

Human glioma cell lines U251, U87, U118, Hs683, and human microglial cell line HMC3 were obtained from Shanghai Fuheng Biotechnology Co., Ltd. Cells were cultured in DMEM (Gibco) supplemented with 10% fetal bovine serum (Gibco). HEB cells were cultured in microglial medium (Gibco). All cell lines were cultured at 37°C and under 5% CO_2_ conditions.

### Lentivirus construction and cell transfection

A lentivirus system encoding shRNA targeting a scrambled sequence and TRAF3IP3 mRNA was used for the knockdown. Sequences targeting TRAF3IP3-sh1 5′-CCATCAAGAAGCCACCCAA-3′ and TRAF3IP3-sh2 5′-CCACGTGCTTCAGTCCAAA-3′ were cloned into the PGIPZ-puro. The coding sequence of human TRAF3IP3 was synthesized and subcloned into a PCDH vector to construct overexpression plasmids. TRAF3IP3-F: AGCGAATTCGCCACCatgatcagcccagacccca; TRAF3IP3-R: GGATCCGATTTAAATtcagatcatcaggttgtctt. The integrity of the plasmid constructs was confirmed by DNA sequencing. Together with the packaged plasmids, the constructed plasmids were transfected into U118 or U251 cells. After 48 h, the supernatant containing the target lentivirus was collected. Lentivirus infection was performed on cells at 80% confluency with a multiplicity of infection (MOI) of 50. Cells were used for downstream assays or transplantation 72 h after infection.

### Cell proliferation assay

The Cell Counting Kit-8 (CCK-8, BBI Life Sciences, E606335-0500) was used to assess the cell proliferation rate. For the cell proliferation assay, U251 cells were infected with lentivirus containing TRAF3IP3 shRNA or scramble shRNA and seeded into 24-well plates. U118 cells were infected with lentivirus containing the PCDH vector or an empty vector. After 96 h, U251 or U118 cells were digested with 0.25% trypsin (Gibco, 25200-056). Cell suspensions were seeded at a density of 3×10^3^ cells/well in 96-well culture plates with three identical wells as duplicate wells, followed by overnight incubation in a 5% CO_2_ humidified incubator at 37°C. After removing the medium, 10 μL of CCK-8 solution was added, and cells were incubated for 1 h at 37°C under 5% CO_2_ conditions. Absorbance was measured at 450 nm using an ELISA plate reader (EPOCH2, BioTek, USA). The OD450 value is inversely proportional to the degree of cell proliferation. Each group comprised three duplicate wells, and the assays were independently performed three times.

### Wound healing assay

Cells (1 ×10^5^/600 μL/well) were seeded in 12-well plates and cultured overnight at 37°C to form a confluent monolayer. Then, an artificial wound was made in the monolayer with a 1 mL pipette tip and washed three times with PBS. Cell activity was recorded every 24 h using an inverted microscope. The test was completed after 48 h. The wound was analyzed using the ImageJ software (version 1.80, National Institute of Health, Bethesda, MD, USA).

### Transwell assay

Transwell invasion assays were performed using Transwell chambers with Matrigel (354480, CORNING). Briefly, 600 μL of 10% FBS-containing medium was placed in the lower chamber, and 2×10^4^ cells suspended in 100 μL serum-free medium were seeded into the upper chamber for 24 h. After 24 h, cells migrated through the Matrigel were fixed with 4% paraformaldehyde (A500684, BBI Life Sciences, China), stained with 0.1% crystal violet (E607309, BBI Life Sciences, China), and counted under a microscope.

### Animal models

Twelve female BALB/c nude mice at 6 weeks of age, weighing 18–22 g, were obtained from Shanghai Jihui Experimental Animal Feeding Co., LTD (Shanghai, China) and bred in a special pathogen-free (SPF) grade laboratory at Tongji University. The mice were housed under a 12:12 h light:dark cycle environment, with *ad libitum* access to food and water. All experiments were approved by the Animal Care and Use Committee of Tongji University. A total of 5×10^4^ U118-GFP-expressing cells or U118 TRAF3IP3-GFP-expressing cells were injected *in situ* into the right caudate nucleus of BALB/c nude mice (six mice per group). Images were captured 1 week after injection and 2 weeks later using a Caliper Life Sciences camera (Ivis Lumina Xr).

### Immunohistochemistry

Paraffin-embedded sections of tumor tissues from nude mice were de-paraffinized using different concentrations of ethanol (100, 95, 85, and 75%) and rehydrated with deionized water. These slices were then immersed in EDTA solution (pH 9.0, G1203, Servicebio) and heated, maintaining the temperature between 95°C and 100°C for 30 min. After washing with PBS, the sections were incubated with a 3% hydrogen peroxide solution for 25 min. The sections were then incubated with primary antibodies targeting Ki67 (1:1000, GB111141, Servicebio) and PCNA (1:500, 10205-2-AP, Proteintech) overnight at 4°C and then stained using HRP-labeled goat anti-rabbit IgG or anti-mouse IgG (Servicebio GB23303).

### Statistical analysis

All data are expressed as the means ± standard deviations (SDs). Comparisons between two groups were analyzed using the Student’s *t*-test. Comparisons among multiple groups were made using one-way analysis of variance (ANOVA), followed by Dunnett’s test. Statistical significance was set at p < 0.05, p < 0.01, or p < 0.001.

## Results

### DEGs in gliomas

Using the *limma* package, we analyzed the DEGs in gliomas in the three datasets and obtained a set of genes whose expressions were significantly upregulated or downregulated in glioma tissues (**Figures S1A–C**). In addition, we performed a cluster analysis of the expression values of DEGs using the three datasets. The results indicated that the DEGs in each group could distinguish tumor tissues from normal tissues (**Figures S1D–F**).

**Figure 1:**
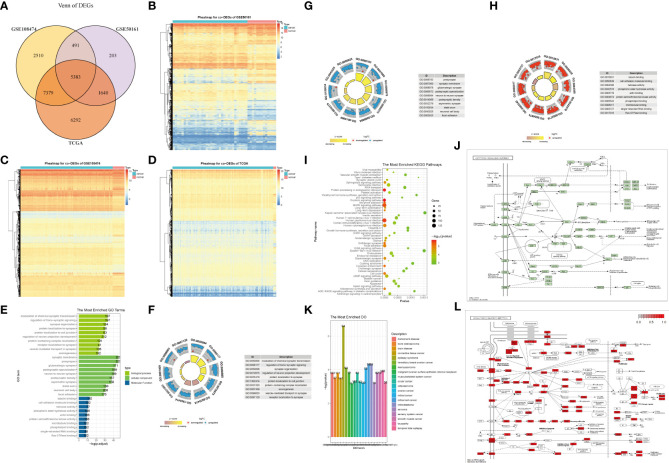
Differentially expressed genes(DEGs) and enrichment analysis in glioma. **(A)** Screening of the intersection of DEGs in the three datasets yielded 5,383 DEGs shared between the three datasets. **(B–D)** DEGs can distinguish tumor tissues from normal tissues in each grouping, which has significant representativeness. **(E–H)** GO function enrichment analysis on DEGs and enrichment of BP, CC, MF. **(I, J, L)** KEGG pathway enrichment analysis. **(K)** GO enrichment analysis.

Subsequently, we screened the intersection of DEGs in the three datasets (**Figure 1A**), obtaining 5383 DEGs shared by the three datasets. To verify the impact of DEGs on clinical diagnosis, we used DEGs and data groupings to plot a classification heat map (**Figures 1B–D**) and determined that DEGs can distinguish diseased samples from normal specimens.

### Screening of key genes

To identify potential genes that could serve as biomarkers of glioma prognosis, we first performed a GO analysis of the DEGs. DEGs were mainly enriched in the modulation of chemical synaptic transmission and regulation of transsynaptic signaling, synaptic membrane, presynaptic, tubulin binding, cell adhesion molecule binding, among other biological processes, cell components, and molecular functions (**Figures 1E–H**, **Table 2**). Next, KEGG analysis of the DEGs was performed. The results showed that DEGs were enriched in the oxytocin signaling pathway, protein processing in the endoplasmic reticulum, human papillomavirus infection, cell cycle, and other biological pathways (**Figures 1I, J, L**). In addition, the GO analysis results showed that DEGs were significantly enriched in epilepsy syndrome, retinoblastoma, retinal cell carcinoma, hereditary breast cancer, and other diseases (**Figure 1K**).

**Table 2:** DEG enrichment analysis between glioma tissue and tumor tissue.

ONTOLOGY	ID	Description	p-value adjusted
BP	GO:0050804	modulation of chemical synaptic transmission	4.32648E-15
BP	GO:0099177	regulation of trans-synaptic signaling	4.32648E-15
BP	GO:0050808	synapse organization	3.81915E-14
BP	GO:0035418	protein localization to synapse	7.88689E-14
BP	GO:1902414	protein localization to cell junction	7.88689E-14
BP	GO:0010975	regulation of neuron projection development	2.43698E-13
BP	GO:0031503	protein-containing complex localization	3.40863E-11
BP	GO:0097120	receptor localization to synapse	4.53789E-11
BP	GO:0099003	vesicle-mediated transport in synapse	1.09306E-10
BP	GO:0007409	axonogenesis	2.12606E-10
CC	GO:0097060	synaptic membrane	1.71269E-20
CC	GO:0098793	presynapse	1.85301E-20
CC	GO:0098978	glutamatergic synapse	3.9456E-19
CC	GO:0099572	postsynaptic specialization	1.65662E-18
CC	GO:0098984	neuron to neuron synapse	2.80624E-18
CC	GO:0014069	postsynaptic density	3.47796E-17
CC	GO:0032279	asymmetric synapse	4.71506E-17
CC	GO:0150034	distal axon	3.20601E-14
CC	GO:0043025	neuronal cell body	4.92734E-14
CC	GO:0005925	focal adhesion	2.7836E-13
MF	GO:0015631	tubulin binding	4.25916E-06
MF	GO:0050839	cell adhesion molecule binding	2.42145E-05
MF	GO:0004386	helicase activity	2.42145E-05
MF	GO:0042578	phosphoric ester hydrolase activity	6.35698E-05
MF	GO:0003779	actin binding	9.3847E-05
MF	GO:0004674	protein serine/threonine kinase activity	0.000107552
MF	GO:0008017	microtubule binding	0.000220913
MF	GO:0005543	phospholipid binding	0.000220913
MF	GO:0003727	single-stranded RNA binding	0.000220913
MF	GO:0017016	Ras GTPase binding	0.000259822
KEGG	hsa04921	Oxytocin signaling pathway	0.000289784
KEGG	hsa04141	Protein processing in endoplasmic reticulum	0.000289784
KEGG	hsa05165	Human papillomavirus infection	0.000289784
KEGG	hsa04010	MAPK signaling pathway	0.000289784
KEGG	hsa05032	Morphine addiction	0.000354324
KEGG	hsa04727	GABAergic synapse	0.000354324
KEGG	hsa04713	Circadian entrainment	0.000354324
KEGG	hsa04110	Cell cycle	0.000354324
KEGG	hsa04925	Aldosterone synthesis and secretion	0.00037778
KEGG	hsa04730	Long-term depression	0.00037778
DO	DOID:1826	epilepsy syndrome	0.000216853
DO	DOID:768	retinoblastoma	0.011280615
DO	DOID:771	retinal cell cancer	0.011280615
DO	DOID:5683	hereditary breast ovarian cancer	0.011280615
DO	DOID:4645	retinal cancer	0.011280615
DO	DOID:680	tauopathy	0.011280615
DO	DOID:1967	leiomyosarcoma	0.011280615
DO	DOID:4230	smooth muscle cancer	0.011280615
DO	DOID:0060116	sensory system cancer	0.011280615
DO	DOID:2174	ocular cancer	0.011280615

**Figure 2:**
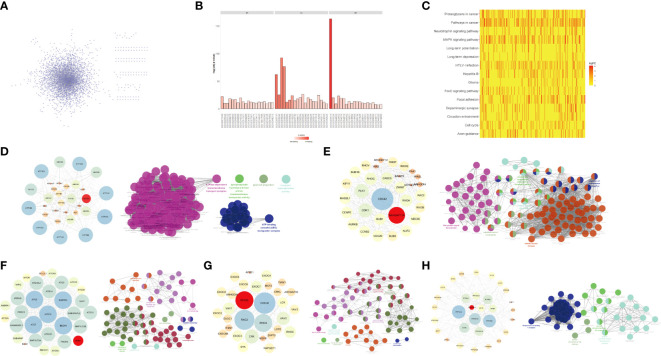
Key genes and their interaction networks. **(A)** The protein-protein interaction (PPI) network related to DEGs. **(B)** GO enrichment analysis of DEGs-related PPI network. **(C)** KEGG enrichment analysis of the PPI network associated with DEGs. **(D)** *ABCB8* gene subnet and its function. **(E)** *ARHGAP11A* gene subnet and its function. **(F)** *WIPI1* gene subnet and its function. **(G)** *RHOQ* gene subnet and its function. **(H)** *TRAF3IP3* gene subnet and its function.

To identify the impact of genes that could serve as glioma prognosis predictors at various stages of glioma development, we downloaded the PPI information from the HPRD database, removing the isolation data nodes, loop interactions, repeated interactions, etc., and extracted the PPI network related to DEGs in the network (**Figure 2A**). To determine PPI networks closely related to DEGs in gliomas, we extracted at least 25 DEGs that interacted with non-DEGs as subnets (**Figure 2B**). We visualized the network with Cytoscape using the cytoHubba plug-in roots to extract the interaction subnet of the top 15 genes. Genes in the PPI subnet were significantly enriched in cancer pathways, MAPK signaling pathways, hepatitis B, pancreatic cancer, colorectal cancer, and other pathways (**Figure 2C**).

By analyzing the glioma-related DEGs and PPI subnets, we obtained five key genes: *TRAF3IP3*, *WIPI1*, *ARHGAP11A*, *ABCB8*, and *RHOQ*. To accurately analyze the mechanism of each key gene, we extracted the PPI subnet of each key gene from the STRING database. We used Cytoscape to visualize the critical gene subnets and used the ClueGO plug-in for functional enrichment analysis (**Figures 2D–H**).

### *TRAF3IP3* may serve as an important indicator for glioma diagnosis and prognosis

**Figure 3:**
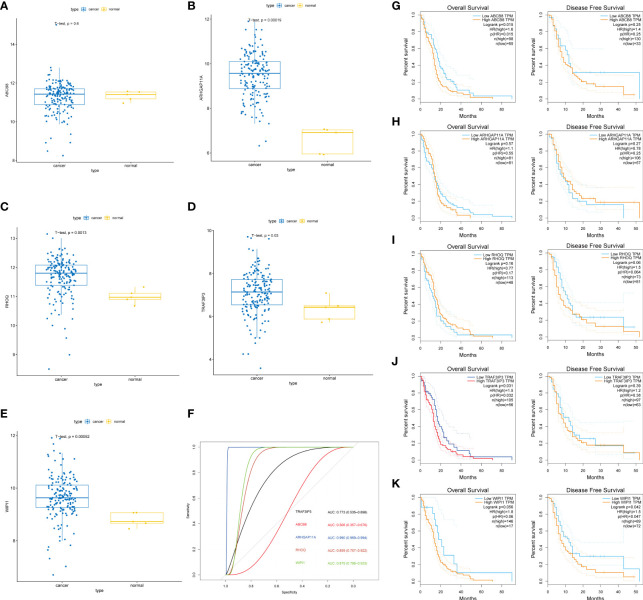
Key genes and their prognostic value. **(A–E)** The differential expression of five key genes in normal and tumor tissue. **(F)** ROC curve and AUC value of five key genes in normal and tumor tissue. **(G–K)** The impact of the expression of the five key genes in tumor tissue on the OS and DFS of patients and their significance.

To determine the value of the key genes, we analyzed their differential expression in normal and tumor tissues (**Figures 3A–E**) and classified the expression levels (**Figure 3F**). The results showed that *TRAF3IP3*, *WIPI1*, *ARHGAP11A*, and *RHOQ* were significantly differentially expressed in normal and tumor tissue, displaying good classification performance. As a result, TRAF3IP3 may serve as an important indicator for glioma diagnosis. We analyzed the impact of key genes on the overall and disease-free survival time (**Figures 3G–K**). The results showed that *TRAF3IP3* and *ABCB8* expression significantly impacted the OS of patients, whereas *WIPI1* expression had a significant impact on the disease-free survival of patients. Therefore, TRAF3IP3 may be an important prognostic factor in gliomas.

### GSEA of TRAF3IP3-related DEGs

**Figure 4:**
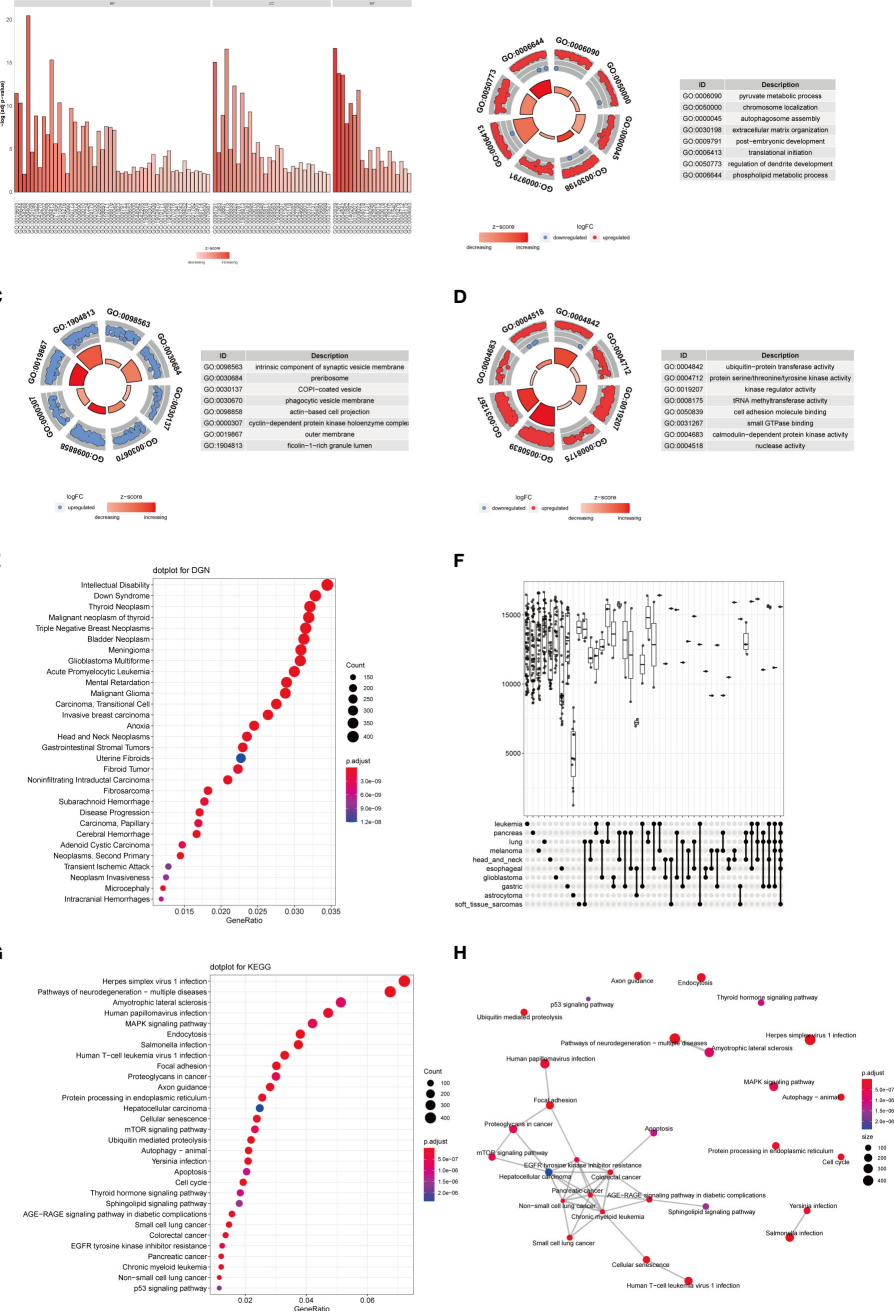
TRAF3IP3-related differential expression gene enrichment analysis. **(A–D)** GO function enrichment analysis using the differentially expressed genes between the TRAF3IP3-high expression group and TRAF3IP3-low expression group, displaying BP, CC, MF. **(E)** DGN enrichment analysis. **(F)** NCG enrichment analysis. **(G)** KEGG enrichment analysis. **(H)** The connection between the enriched KEGG pathways.

To further analyze the influence of *TRAF3IP3* expression in glioma, we identified DEGs between the TRAF3IP3 high- and TRAF3IP3 low-expression groups (**Figures S2A, B**). We performed functional annotations of the DEGs between the two groups (**Table 3**). The results showed that the DEGs between the TRAF3IP3 high- and low-expression groups were significantly enriched in autophagy, autophagy mechanisms, proteasome protein catabolism, proteasome-mediated ubiquitin-dependent protein catabolism, mitochondrial matrix, cell-substrate connection, interneuron synapse, cell adhesion molecule binding, protein serine/threonine kinase activity, and ubiquitin-like protein ligase binding as GO terms (**Figures 4A–D**). In addition, annotations in the DEGs between the TRAF3IP3 high-and low-expression groups were significantly enriched in herpes simplex virus type 1 infection, *Salmonella* infection, ubiquitin-mediated proteolysis, T cell leukemia virus type 1 infection, and neurodegeneration changes in a variety of diseases, small cell lung cancer, cell cycle, and other biological pathways (**Figures 4G, H**). *Salmonella* infection is a major public health concern. Infection in humans can be chronic and increase the risk of cancers (20). Ubiquitin-mediated proteolysis is reportedly associated with pancreatic cancer metastasis (21). Human T cell leukemia virus type 1 (HTLV-1) mainly infects CD4^+^ T cells and induces chronic, persistent infection in infected individuals, with some developing adult T cell leukemia/lymphoma (ATL) (22). Glioblastoma is one of the most aggressive and frequent primary brain tumors. This type of glioma expands and infiltrates into the brain, causing neuronal degeneration and neurological decay (23). A previous study has shown that high TUBA1C expression is associated with poor prognosis in patients with glioma. TUBA1C inhibition reduced glioma cell proliferation through cell cycle arrest (24). Thus, TRAF3IP3 may be associated with cancer progression-related pathways. Furthermore, we analyzed the disease gene network (DGN) and the cancer gene network (CGN), wherein the DEGs between the TRAF3IP3 high- and low-expression groups were significantly enriched (**Figures 4E, F**). These DEGs were significantly enriched in glioblastoma, invasive breast cancer, bladder cancer, and other tumors. TRAF3IP3 is involved in marginal zone B lymphocyte development and survival. TRAF3IP3 contributes to MZ B cell survival by upregulating autophagy, thereby promoting the TI-II immune response (25). TRAF3IP3 was associated with the OS of patients with T1-4N0-2M0 in TCGA dataset. TRAF3IP3 can be used as a prognostic signature for OS in patients with lung adenocarcinoma (26). Gastric cancer is one of the leading causes of cancer-associated mortality. Recent studies show that TRAF3IP3 positively correlated with pathological tumor and lymph node stages (27). Therefore, TRAF3IP3 may be involved in glioma progression.

**Table 3:** Enrichment analysis of DEGs between TRAF3IP3-high expression group and TRAF3IP3-low expression group.

ONTOLOGY	ID	Description	p-value adjusted
BP	GO:0006914	autophagy	5.60388E-34
BP	GO:0061919	process utilizing autophagic mechanism	5.60388E-34
BP	GO:0010498	proteasomal protein catabolic process	7.47E-33
BP	GO:0043161	proteasome-mediated ubiquitin-dependent protein catabolic process	7.36614E-30
BP	GO:0010975	regulation of neuron projection development	4.61246E-29
BP	GO:0034660	ncRNA metabolic process	4.64154E-28
BP	GO:0022604	regulation of cell morphogenesis	3.42525E-26
BP	GO:0034470	ncRNA processing	3.42525E-26
BP	GO:0006605	protein targeting	3.42525E-26
CC	GO:0005925	focal adhesion	3.99213E-36
CC	GO:0005759	mitochondrial matrix	3.99213E-36
CC	GO:0030055	cell-substrate junction	3.52855E-33
CC	GO:0098984	neuron to neuron synapse	7.24578E-33
CC	GO:0014069	postsynaptic density	5.63352E-32
CC	GO:0032279	asymmetric synapse	2.17384E-31
CC	GO:0016607	nuclear speck	6.65435E-30
CC	GO:0099572	postsynaptic specialization	1.02123E-29
CC	GO:0031252	cell leading edge	2.52997E-27
CC	GO:0098978	glutamatergic synapse	4.59204E-27
MF	GO:0050839	cell adhesion molecule binding	2.14177E-17
MF	GO:0004674	protein serine/threonine kinase activity	4.57637E-17
MF	GO:0044389	ubiquitin-like protein ligase binding	2.09118E-16
MF	GO:0031625	ubiquitin protein ligase binding	1.10285E-15
MF	GO:0045296	cadherin binding	1.10749E-15
MF	GO:0140098	catalytic activity, acting on RNA	1.10749E-15
MF	GO:0019787	ubiquitin-like protein transferase activity	5.27907E-15
MF	GO:0031267	small GTPase binding	1.76494E-14
MF	GO:0004842	ubiquitin-protein transferase activity	2.69553E-14
MF	GO:0017016	Ras GTPase binding	1.63233E-13
KEGG	hsa05168	Herpes simplex virus 1 infection	7.55E-13
KEGG	hsa04144	Endocytosis	1.08E-11
KEGG	hsa04360	Axon guidance	1.32E-11
KEGG	hsa05132	Salmonella infection	4.8E-11
KEGG	hsa04120	Ubiquitin mediated proteolysis	1.59E-10
KEGG	hsa05166	Human T-cell leukemia virus 1 infection	6.33E-10
KEGG	hsa05022	Pathways of neurodegeneration - multiple diseases	1.54E-09
KEGG	hsa05222	Small cell lung cancer	2.07E-09
KEGG	hsa04510	Focal adhesion	3.42E-09
KEGG	hsa04110	Cell cycle	3.59E-09

**Figure 5:**
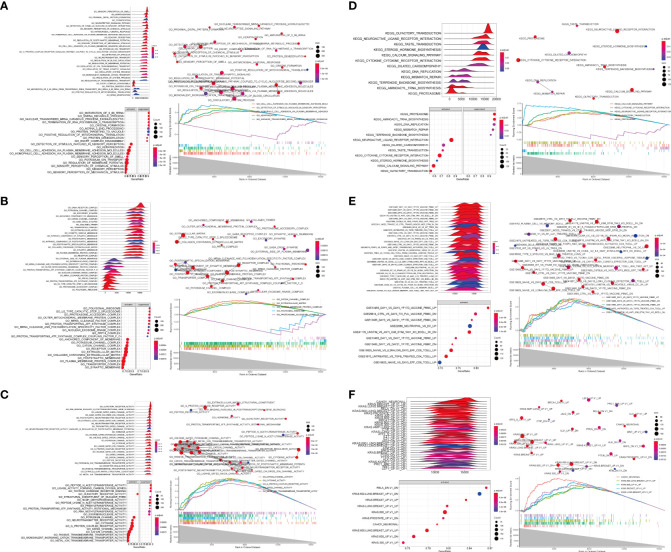
GSEA of differentially expressed genes (DEGs) related to TRAF3IP3. **(A–C)** TRAF3IP3-related DEGs are mainly enriched in terms in KEGG/BP/CC/MF/oncogenic/immunologic, the relationship between each term, and the activation or inhibition of genes in term. **(D–F)** GSEA of DEGs related to TRAF3IP3.

To further identify the functions related to the DEGs in the TRAF3IP3 high-and low-expression groups, we performed enrichment analysis on different GSEA datasets and found that these genes were significantly enriched in neuroactive ligand-receptor interactions, olfactory transmission, proteasome, cytokine–cytokine receptor interaction, and calcium signaling pathway in KEGG biological pathways (**Figure 5A**). They were also significantly enriched in the detection of stimuli in sensory perception, the adhesion of homophilic cells through plasma membrane adhesion molecules, mitochondrial membrane tissue keratinization, sensory perception, and other biological processes (**Figure 5B**); cation channel complex, cytoplasmic ribosomal large subunit, endopeptidase complex, extracellular matrix, mitochondrial ribosomal large subunits, and other cellular components (**Figure 5C**); and in cation channel activity, cytokine activity, gated channel activity, G protein-coupled receptor activity, metal ion transmembrane transport protein activity, and other molecular functions (**Figure 5D**). A biological information analysis suggested that neuroactive ligand-receptor interactions may be associated with glioma (28), which is consistent with our results. Despite all other cells that have the potential to prevent cancer development and metastasis through tumor-suppressor proteins, cancer cells can promote the activity of the ubiquitin-proteasome system (UPS) to degrade tumor-suppressor proteins and avoid apoptosis. As less invasive chemotherapy drugs, UPS inhibitors are increasingly used to alleviate the symptoms of various malignant cancers. Ubiquitin-proteasome is associated with cancer development (29). A previous study demonstrated that the DEGs in glioma were significantly enriched in the cytokine–cytokine receptor interaction, which is consistent with our study (30). We also carried out enrichment analysis on the cancer characteristic gene set and immune characteristic-related gene set (**Figures 5E, F**). The cancer characteristic gene set included PTEN, IL21, KRAS, etc. Loss of the tumor-suppressor molecule PTEN is among the most common molecular dysfunctions associated with glioma malignancy (31). Besides, interleukin-21 (IL21) increases the reactivity of allogeneic human Vγ9Vδ2 T cells against primary glioblastoma tumors. IL-21 increased intracellular granzyme B levels and cytotoxicity of allogeneic human Vγ9Vδ2 T lymphocytes *in vitro* (32). Kirsten rat sarcoma viral oncogene (KRAS) expression exhibited a positive correlation with the ERK pathway. KRAS overexpression promoted glioma proliferation and invasion (33). The immune characteristic-related gene set included IL2, NOD2, IL21, etc. Interleukin-2 and histamine jointly inhibit tumor growth and angiogenesis in malignant glioma (34). A study showed a significant correlation between nucleotide oligomerization domain 2 (NOD2) variants and a higher risk of glioblastoma (35). Therefore, TRAF3IP3 may be associated with glioma progression-related pathways.

### *TRAF3IP3* regulates glioma cell proliferation, migration, and invasion *in vitro*

**Figure 6:**
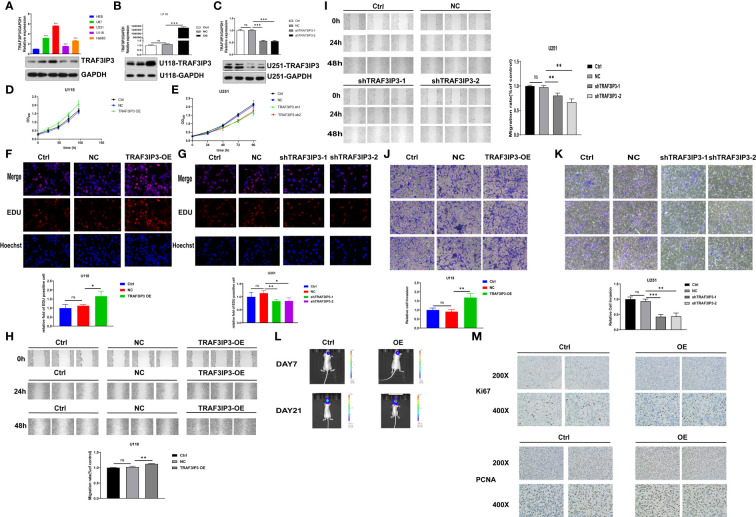
*TRAF3IP3* promotes tumor progression *in vitro* and *in vivo*. **(A)** The levels of TRAF3IP3 in glioma cell lines were detected using qRT-PCR and western blot, *** vs HEB p < 0.001. **(B)** The overexpression efficiency was verified after overexpression of *TRAF3IP3* in U118 glioma cells, ***p < 0.001. **(C)** The knockdown efficiency was verified after knockdown of *TRAF3IP3* in U251 glioma cells, ***p < 0.001. **(D)** Cell proliferation was significantly increased in U118 glioma cells infected with PCDH/TRAF3IP3 vector compared with the NC group and control group, *** p < 0.001. **(E)** CCK-8 assays showed that silencing of TRAF3IP3 expression significantly suppressed cell proliferation of U251 cells compared with the NC group and control group, **p < 0.01. **(F)** EdU assay revealed that overexpression of *TRAF3IP3* in U118 glioma cells significantly promoted cell proliferation, *p < 0.05. **(G)** Knockdown of *TRAF3IP3* in U251 glioma cells significantly reduced cell proliferation, *p < 0.05, **p < 0.01. **(H)** Wound-healing assay showed that the ectopic overexpression of *TRAF3IP3* could obviously enhance the migration rate of U118 cells, **p < 0.01. **(I)** *TRAF3IP3* knockdown in U251 cells had the significant opposite effect, **p < 0.01. **(J)** Ectopic expression of *TRAF3IP3* significantly enhanced the invaded rate of glioma cells, **p < 0.01. **(K)** Silencing of *TRAF3IP3* expression decreased the number of invaded glioma cells, **p < 0.01, ***p < 0.001. **(L)** Fluorescence imaging experiment in nude mice showed that there was no statistically significant difference in tumor growth in mice 7 days after injection of TRAF3IP3-overexpressed cells (n=5, experimental group vs. control group). Tumor growth showed significant statistical difference in mice 21 days after injection of TRAF3IP3-overexpressed glioma cells, ** vs. Ctrl, p < 0.01, n=5. **(M)** Overexpression of TRAF3IP3 in U118 cells promotes increase in the expression levels of proliferation-related biomarkers KI67 and PCNA. ns, Not Statistically Significant.

To investigate the role of *TRAF3IP3* in glioma development, TRAF3IP3 expression in glioma cell lines was detected using qRT-PCR and western blot (**Figure 6A**). We showed that TRAF3IP3 expression was upregulated in glioma cell lines compared to human glial cell line HEB. These results indicate that *TRAF3IP3* may be involved in glioma development.

To explore the biological function of TRAF3IP3 in tumorigenesis, qRT-PCR and western blot were used to verify *TRAF3IP3* expression after its overexpression in U118 cells (**Figure 6B**). Similarly, we detected TRAF3IP3 expression in glioma cells U251 after *TRAF3IP3* knockdown. The western blot showed partial knockdown (50%) of TRAF3IP3 (**Figure 6C**).

To explore the potential role of TRAF3IP3 in tumorigenesis, we evaluated its effects on the growth of glioma cells *in vitro*. CCK-8 assays revealed that TRAF3IP3 overexpression significantly increased the proliferation of U118 cells compared to the NC and control group (**Figure 6D**). However, partial knockdown of TRAF3IP3 in U251 glioma cells significantly suppressed U251 cell proliferation compared to controls (**Figure 6E**). Collectively, these data suggest that *TRAF3IP3* is involved in the proliferative ability of gliomas. In addition, we used the EdU assay to verify that *TRAF3IP3* overexpression in U118 glioma cells significantly promoted cell proliferation (**Figure 6F**). In contrast, *TRAF3IP3* knockdown in U251 glioma cells significantly reduced cell proliferation (**Figure 6G**).

To evaluate the effect of *TRAF3IP3* on glioma cell migration, we performed a wound healing assay. The wound healing assay results showed that ectopic overexpression of *TRAF3IP3* enhanced the migration abilities of U118 cells (**Figure 6H**). Conversely, *TRAF3IP3* knockdown in U251 cells had the opposite effect (**Figure 6I**). Taken together, these results indicated that *TRAF3IP3* promotes glioma cell migration.

Transwell assays were performed to validate the effect of *TRAF3IP3* overexpression and silencing on the migration and invasion abilities of glioma U118 and U251 cells. As shown in **Figure 6J**, ectopic expression of *TRAF3IP3* significantly enhanced the invasion ability of glioma cells, whereas silencing of *TRAF3IP3* expression decreased the number of invading glioma cells (**Figure 6K**). These results revealed that *TRAF3IP3* promotes glioma cell invasion.

### *TRAF3IP3* promotes glioma cell growth *in vivo*

To further verify the role of TRAF3IP3 in glioma cell growth, cells expressing ectopic TRAF3IP3 and control cells were injected *in situ* into the caudate nucleus of nude mice to generate a xenotransplantation model. Fluorescence imaging experiments in nude mice showed no statistically significant difference in tumor growth in mice 7 days after the injection of TRAF3IP3-overexpressing cells. However, tumor growth significantly differed in mice 21 days after injection of TRAF3IP3-overexpressing glioma cells (**Figure 6L**). Moreover, we detected that the expression of the proliferation-related biomarkers, Ki67 and PCNA, was increased in TRAF3IP3-overexpressing tumors (**Figure 6M**). Taken together, these results indicated that TRAF3IP3 plays a critical role in glioma cell growth.

### *TRAF3IP3* exerts an oncogenic role *via* the ERK pathway in glioma cells

**Figure 7:**
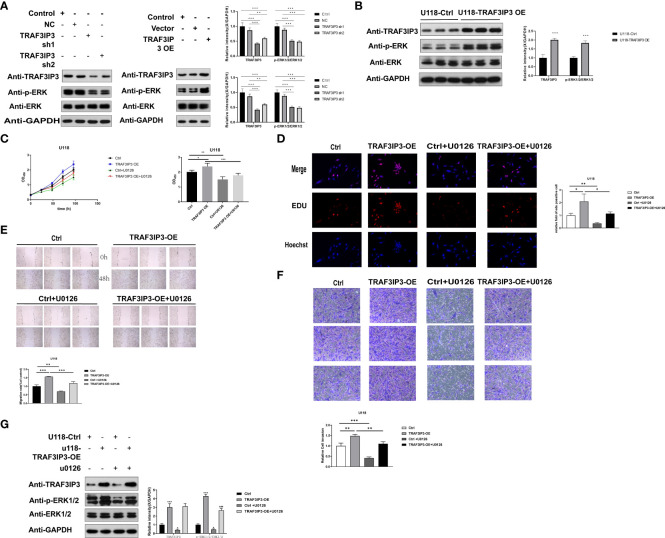
*TRAF3IP3* promotes tumor progression *via* ERK signaling. **(A)** *TRAF3IP3* knockdown in U251 cells decreased the phosphorylation of ERK, whereas *TRAF3IP3* overexpression in U118 cells increased the phosphorylation of ERK. **(B)** *TRAF3IP3* overexpression of U118 cells increased ERK activation. **(C,D)** CCK-8 assay and EdU assay showed that *TRAF3IP3* activated the ERK signaling pathway to promote tumor proliferation, which could be rescued by U0126. **(E)** Wound-healing assay showed that *TRAF3IP3* activated the ERK signaling pathway to promote tumor migration, which could be rescued by U0126. **(F)** Transwell assays showed that *TRAF3IP3* activated the ERK signaling pathway to promote tumor invasion, which could be rescued by U0126. **(G)** The effect of *TRAF3IP3* overexpression in activating ERK signaling pathway could be rescued by U0126. *p < 0.05, **p<0.01, ***p<0.001, ### p<0.001.

Bioinformatics analysis showed that DEGs were enriched in the MAPK signaling pathway in GBM. To explore the downstream signaling pathway of TRAF3IP3, we detected the changes in downstream signaling pathways in U118 and U251 cells after *TRAF3IP3* overexpression or knockdown. *TRAF3IP3* knockdown in U251 cells decreased ERK phosphorylation, whereas *TRAF3IP3* overexpression increased ERK phosphorylation (**Figure 7A**). TRAF3IP3 overexpression in U118 cells increased ERK activation (**Figure 7B**). Thus, we speculated that the ERK signaling pathway might be associated with the oncogenic function of TRAF3IP3 in glioma cells.

To confirm the association between *TRAF3IP3* and the ERK pathway and to identify whether the ERK pathway is involved in *TRAF3IP3*-mediated glioma proliferation, migration, and invasion *in vitro*, U118 cells transfected with the PCDH/TRAF3IP3 vector were treated with or without the ERK pathway inhibitor U0126. We found that U0126 treatment significantly inhibited cell proliferation, migration, and invasion (**Figures 7C–F**) in PCDH/TRAF3IP3-transfected glioma cells. Quantitative western blot analysis revealed that the ERK pathway was inhibited when TRAF3IP3 was knocked down alone, whereas TRAF3IP3 rescue by overexpression resulted in ERK pathway activation (**Figure 7G**). In TRAF3IP3-overexpressing cells, the addition of U0126 led to the reversal of ERK pathway activation. This change was also found to be statistically significant when compared to the U0126 control group. These results indicated that *TRAF3IP3* functions as an oncogene, possibly *via* ERK pathway activation in glioma cells.

## Discussion

Glioma is one of the deadliest malignant nervous system tumors worldwide (36). Although glioma treatment alternatives (i.e., chemotherapy and radiotherapy) have advanced in the past decades, metastasis and disease recurrence continue to pose challenges to physicians and patients (37). In addition, the mechanisms underlying the proliferation, migration, and invasion of gliomas have not been clarified. Hence, identifying novel key targets and potential mechanisms involved in cancer cell proliferation and migration is critically important.

Data obtained from GeneCards indicated that TRAF3IP3 is located on the human chromosome Chr1q32.2, mainly localized in the nucleus. Thus, it may function as an adapter molecule that regulates TRAF3-mediated JNK activation. Previous studies have confirmed that TRAF3IP3 is mainly involved in T lymphocyte development and maturation. TRAF3IP3 is reportedly involved in antiviral immunity (38). Few studies have investigated its role in tumor progression. Recent studies have established a key role of TRAF3IP3 in melanoma proliferation, invasion, and metastasis. TRAF3IP3 expression is significantly higher in melanoma than in normal tissues (10). Yang et al. previously reported that high TRAF3IP3 expression in patients with glioma might be associated with poorer prognoses (39). Compared to the study by Yang et al., our study is more in-depth and detailed with regard to bioinformatic analyses. We added a layer-by-layer screening process of genes from the total glioma dataset, additionally including two GEO datasets for analysis, which was in line with the logic of discovering the function of candidate genes. Overall, we identified five key genes using bioinformatic analyses and further verified gene functions through a large number of molecular experiments, ultimately selecting TRAF3IP3 for further study. Finally, the greatest difference between our study and that published by Yang et al. is that we not only confirmed TRAF3IP3 as a key gene through bioinformatic analysis but also verified its function. In this study, bioinformatic analysis of the prognostic value of TRAF3IP3 in glioma was performed using high-throughput data from TCGA/GEO.

First, we analyzed DEGs between tumor and normal tissues from GSE50161, GSE108474, and TCGA databases to investigate the gene expression profiles in GBM tumor tissue relative to normal tissue, obtaining 5,383 DEGs common to the three datasets. We found that these DEGs could distinguish between tumor and normal tissues.

Furthermore, we analyzed the functional enrichment of DEGs through GO/KEGG analyses and found that DEGs were mainly enriched in the regulation of chemical synaptic transmission and transsynaptic signaling, in the biological process of synaptic organization, synaptic protein localization, protein localization at cell junctions, and regulation of neuron projection development. Next, we analyzed the DEGs and PPI subnets related to glioma and obtained five key genes: *TRAF3IP3*, *WIPI1*, *ARHGAP11A*, *ABCB8*, and *RHOQ*. By analyzing the expression of these genes in normal and tumor tissue, we found that they were significantly differentially expressed in normal and tumor tissue, reflecting a clear predictive value for glioma. Tianfei Ran et al. have demonstrated that WIPI1 promotes osteosarcoma cell proliferation by inhibiting CDKN1A expression (40). ARHGAP11A, a prognostic biomarker, is correlated with immune infiltrates in gastric cancer. In addition, high ARHGAP11A expression significantly correlated with a better prognosis in gastric cancer (41). However, the role of ARHGAP11A in glioma remains unclear. ABCB8 is overexpressed in phenotypically aggressive clear-cell renal cell carcinoma and plays a role in promoting the growth of renal clear cell carcinoma (42). High expression of RHOQ promoted colon adenocarcinoma cell growth and was associated with a lower survival rate in patients with colon adenocarcinoma (43). We also analyzed the influence of key genes on OS and disease-free survival and found that *TRAF3IP3* and *ABCB8* expression had a significant impact on the OS of patients. Furthermore, *WIPI1* expression significantly impacted the disease-free survival of patients. Thus, *TRAF3IP3* may serve as an important indicator for glioma diagnosis and prognosis.

Finally, we confirmed that *TRAF3IP3* might serve as a potential prognostic biomarker for glioma. *TRAF3IP3* expression was higher in glioma cell lines than in normal cells and tumor tissues than in tumor-adjacent specimens. Furthermore, we showed that ectopic expression of *TRAF3IP3* enhanced cancer growth, proliferation, and migration, whereas silencing *TRAF3IP3* inhibited the growth, motility, and metastasis of glioma cells. *In vivo*, remarkable promotion of tumor growth was observed following *TRAF3IP3* overexpression. Our findings suggested that *TRAF3IP3* plays an oncogenic role in glioma progression by promoting cell growth.

Previous studies have shown that *TRAF3IP3* plays a vital role in IFN-mediated antiviral innate immunity and participates in RIG-I-MAVS-mediated antiviral signaling (22). However, it is unclear which signaling pathway is activated by *TRAF3IP3* in glioma and whether other crosstalk pathways are involved. According to the GSEA analysis of TRAF3IP3-related DEGs, the ERK signaling pathway was selected to verify whether it participated in glioma progression. Our study found that *TRAF3IP3* may be involved in activating the ERK signaling pathway to promote proliferation, migration, and invasion of glioma cells. *TRAF3IP3* knockdown in U118 cells decreased ERK phosphorylation, whereas *TRAF3IP3* overexpression increased ERK phosphorylation. TRAF3IP3 overexpression in U118 glioma cells enhanced the proliferation, migration, and invasion abilities of glioma cells and ERK phosphorylation, which could be rescued by the ERK signaling pathway inhibitor U0126. In summary, our results indicated that *TRAF3IP3* expression was associated with glioma prognosis and concluded that *TRAF3IP3* could play an oncogenic role in glioma. Our findings provide additional insight into using *TRAF3IP3* as a target to control tumor growth and development.

Although our findings have improved our understanding of the relationship between *TRAF3IP3* and glioma, the study has a few limitations. First, to fully clarify the specific role of *TRAF3IP3* in glioma development, all clinical factors should be considered, including the details of patient treatment. However, the lack of such information in public databases or inconsistencies in treatment can lead to inaccurate results. Second, the sample size was small, which can also cause inaccuracies. Third, we selected two glioma cell lines for cell-based experiments: one cell line for overexpression and another for the knockdown. It would have been ideal to use both cell lines concurrently in all experiments, and we plan to conduct these experiments as part of our ongoing work in the future. In addition, as primary cells are more representative, it would have been preferable to use primary cells for functional experiments. We will address these issues in our future studies. Finally, the ERK pathway was inhibited when TRAF3IP3 was knocked down alone, but it was activated in rescue experiments when TRAF3IP3 was overexpressed. Group 4 was defined by adding U0126 into TRAF3IP3-overexpressing cells, which led to the reversal of ERK pathway activation. Compared with TRAF3IP3 overexpression alone, adding U0126 when TRAF3IP3 was overexpressed (group 4) reversed the activation of the ERK pathway. There was also a significant difference between group 4 and the groups treated with the inhibitor U0126 alone, suggesting that the effects observed in group 4 are not merely the effects of the inhibitor U0126. Although we identified TRAF3IP3 as a key gene related to glioma progression, the identity of the molecule(s) that TRAF3IP3 interacts with to regulate glioma cell proliferation, migration, and invasion through ERK signaling remains unclear. Therefore, further studies on the underlying mechanisms of *TRAF3IP3* in gliomas are warranted.

In conclusion, *TRAF3IP3* may predict poor prognosis and play important roles during glioma development. Moreover, the ERK signaling pathway may be a pivotal *TRAF3IP3*-regulated pathway in gliomas. Nonetheless, further studies should be performed to elucidate the mechanisms underlying these processes.

## Data availability statement

The original contributions presented in the study are included in the article/**Supplementary Material**. Further inquiries can be directed to the corresponding authors.

## Ethics statement

The animal study was reviewed and approved by Institute Research Ethics Committee of the tenth people’s hospital of shanghai.

## Author contributions

Conception and design of the study by LG and X-ZC. Implementation and analysis of *in vitro* data by QL, Z-LS, and FX. Acquisition, analysis, and interpretation of *in vivo* data by J-JQ. Writing or reviewing the manuscript by ZC, X-PK, Z-RC, and Z-YX. All authors contributed to the article and approved the submitted version.

## Funding

This work was supported by scientific research funds from the key departments of the Shanghai Tenth People’s Hospital.

## Acknowledgments

We would like to thank Professor Liang Gao and Xian Zhen Chen for their assistance with data analysis.

## Conflict of interest

The authors declare that the research was conducted in the absence of any commercial or financial relationships that could be construed as a potential conflict of interest.

## Publisher’s note

All claims expressed in this article are solely those of the authors and do not necessarily represent those of their affiliated organizations, or those of the publisher, the editors and the reviewers. Any product that may be evaluated in this article, or claim that may be made by its manufacturer, is not guaranteed or endorsed by the publisher.

## Supplementary material

Supplementary Figure 1 Differentially expressed genes (DEGs) in gliomas. **(A–C)** The overall distribution of gene expression differences was visualized using volcano plots. **(D–F)** The DEGs of each group can distinguish tumor tissues from normal tissues well.

Supplementary Figure 2 Differentially expressed genes (DEGs) associated with TRAF3IP3 in gliomas. **(A)** Differential analysis between TRAF3IP3 high expression group and TRAF3IP3 low expression group in glioma tissue. **(B)** DEGs between the TRAF3IP3 high and TRAF3IP3 low expression groups in the glioma tissues were divided into two groups. DEGs can distinguish between the two groups.

## Abbreviations

DEG, differentially expressed gene; DGN, disease gene network; GO, Gene Ontology; GSEA, Gene set enrichment analysis; KEGG, Kyoto Encyclopedia of Genes and Genomes; MOI, multiplicity of infection; OS, overall survival; PPI, protein–protein interaction; SPF, special pathogen-free; TCGA, The Cancer Genome Atlas.
