# Supplementary figures and images for: TRAF3IP3 promotes glioma progression through the ERK signaling pathway
Source: Front Oncol. 2022 Sep 16;12:776834. doi: 10.3389/fonc.2022.776834 (PMC9523251; doi:10.3389/fonc.2022.776834)

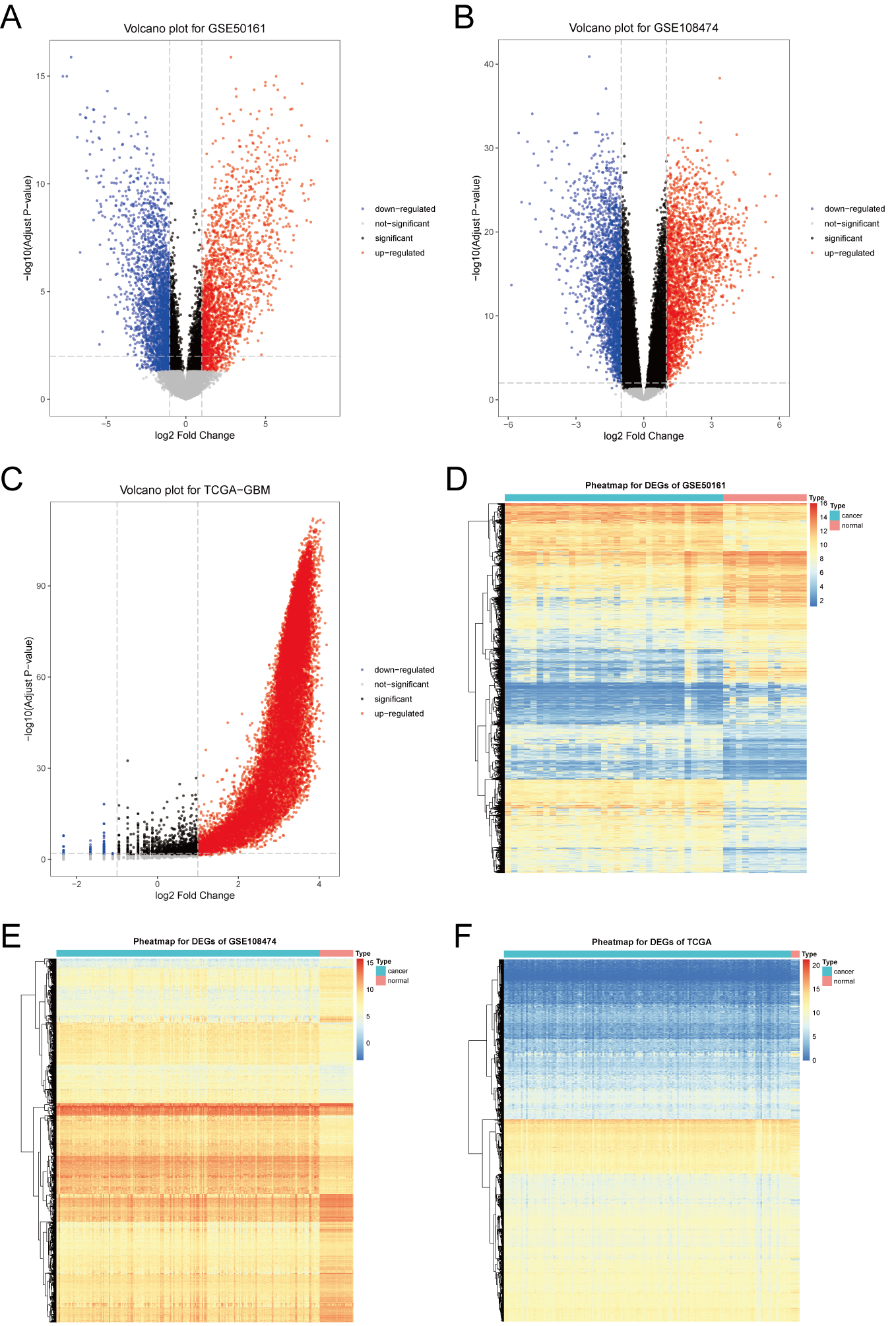

Supplement: Supplementary Figure 1 — Differentially expressed genes (DEGs) in gliomas. (A–C) The overall distribution of gene expression differences was visualized using volcano plots. (D–F) The DEGs of each group can distinguish tumor tissues from normal tissues well. [file Image_1.tif]

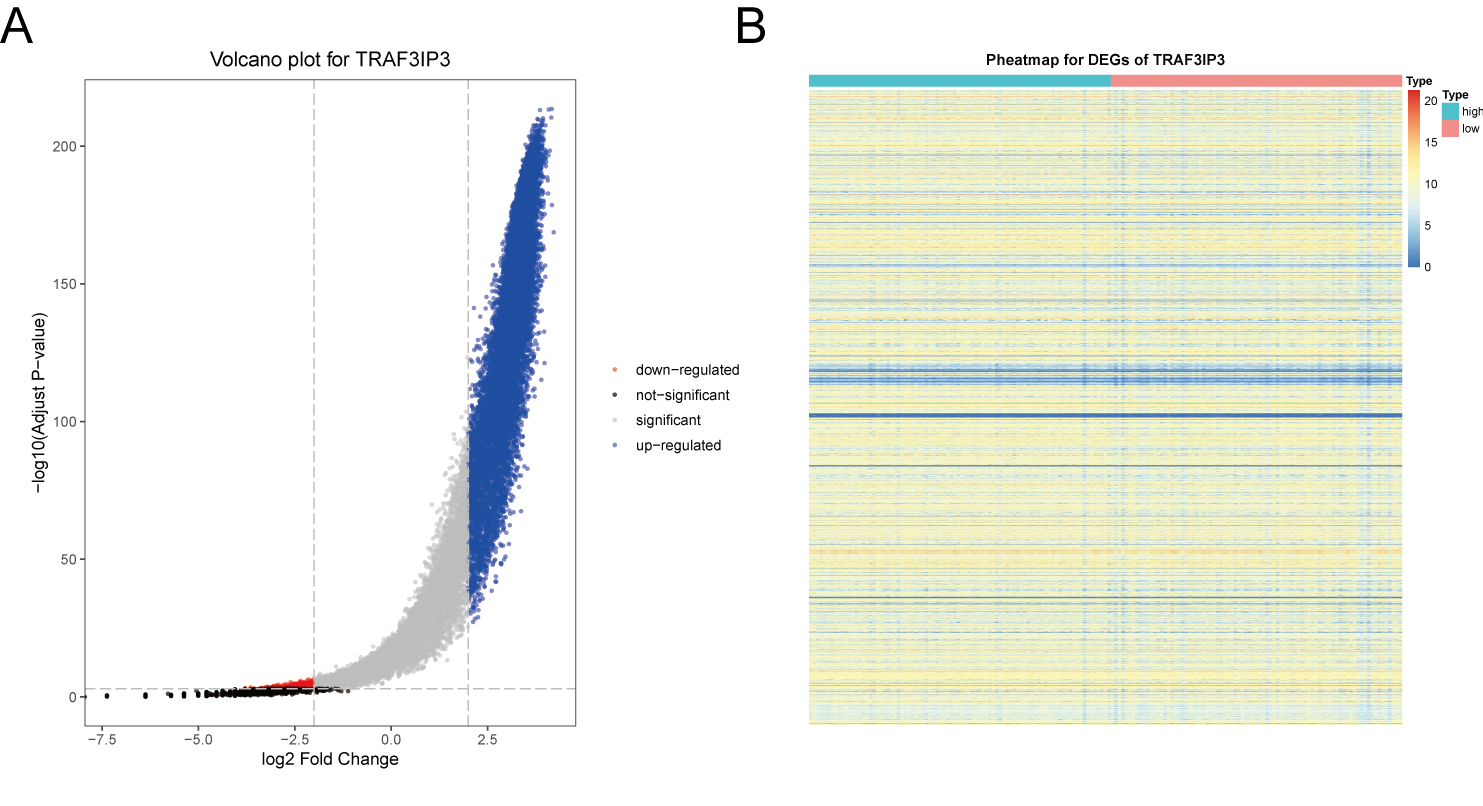

Supplement: Supplementary Figure 2 — Differentially expressed genes (DEGs) associated with TRAF3IP3 in gliomas. (A) Differential analysis between TRAF3IP3 high expression group and TRAF3IP3 low expression group in glioma tissue. (B) DEGs between the TRAF3IP3 high and TRAF3IP3 low expression groups in the glioma tissues were divided into two groups. DEGs can distinguish between the two groups. [file Image_2.tif]
